# Antimicrobial stewardship at a tertiary center in Portugal: insights from prescribers

**DOI:** 10.1017/ash.2025.10263

**Published:** 2025-12-22

**Authors:** André Valois, Mariana Salvado de Morais, Lúcia Ribeiro Dias, Francisco Almeida, Mariana Guedes, Paulo Andrade, Nuno Rocha-Pereira

**Affiliations:** 1 Department of Biomedicine, Faculty of Medicine, University of Porto, Porto, Portugal; 2 Clinical Pharmacology Unit, São João Local Health Unit (ULS São João), Porto, Portugal; 3 Internal Medicine Department, São José Local Health Unit (ULS de São José), Lisbon, Portugal; 4 Department of Medicine, Faculty of Medicine, University of Porto, Porto, Portugal; 5 Infectious Diseases Department, São João Local Health Unit (ULS São João), Porto, Portugal; 6 Infection and Antimicrobial Resistance Control and Prevention Unit, Hospital Epidemiology Centre, São João Local Health Unit (ULS São João), Porto, Portugal; 7 Department of Public Health and Forensic Sciences and Medical Education, University of Porto, Porto, Portugal

## Abstract

**Objective:**

To evaluate physician engagement with an antimicrobial stewardship program (ASP) at a tertiary hospital and identify areas for improvement in the delivery of stewardship interventions.

**Design:**

Cross-sectional survey study.

**Setting:**

A 1200-bed tertiary care public hospital in Portugal.

**Participants:**

Physicians with antibiotic prescribing privileges in inpatient settings. All physicians with active institutional e-mail addresses were invited to participate.

**Methods:**

An anonymous web-based questionnaire was administered between June and December 2024. The survey addressed six domains: (1) antibiotic knowledge; (2) awareness and use of local prescribing protocols; (3) access to antimicrobial use and resistance reports; (4) awareness of antibiotic restriction policies; (5) use of informal consultations with the stewardship team; and (6) participation in scheduled multidisciplinary case discussions.

**Results:**

A total of 154 prescribing physicians responded (response rate: 10%), including specialists (75.3%) and residents (24.0%). Most respondents were aware of institutional protocols (78.6%), but 66.9% reported rarely or never consulting them, citing accessibility and reliance on personal knowledge as key barriers. Participation in case discussions was limited (25.3%) but viewed as highly useful. Awareness of restrictive antibiotic policies was low; however, 69.5% indicated that these policies influenced their prescribing behavior. Respondents expressed strong interest in regular feedback on antimicrobial use and resistance trends.

**Conclusions:**

Key areas for improvement in ASP implementation include enhancing access to protocols, expanding case-based discussions, clarifying communication around restrictions, and establishing regular feedback mechanisms. These findings may inform efforts to align ASP strategies with clinical realities in similar hospital settings.

## Introduction

Antimicrobial resistance (AMR) is a major public health threat worldwide. Various national and international entities have launched initiatives to prevent its emergence and to promote the rational use of antibiotics.^1,2^ This concern was most recently reaffirmed in the 2024 United Nations High-Level Meeting on AMR,^3^ where Heads of State emphasized the need to strengthen multisectoral responses through antimicrobial stewardship (AMS) and other coordinated efforts.

In Portugal, the National Program for the Prevention and Control of Infections and Antimicrobial Resistance (PPCIRA) was established in 2013^4^ and is considered a national health priority. PPCIRA operates at central, regional (UR-PPCIRA), and local (UL-PPCIRA) levels. As per national legislation, local units must implement an Antibiotic Stewardship Program (ASP) to optimize therapy and reduce ecological impact.

While the design and objectives of ASPs are well established, their influence on clinical decision-making is less well understood. A recent survey conducted at our institution^5^ highlighted significant variability in prescribing practices across departments, shaped by local cultures, hierarchies, and individual attitudes toward stewardship. These findings suggest that ASPs’ impact depends not only on protocols but also on contextual and behavioral factors.

At our tertiary center, the UL-PPCIRA, which was created in 2014, leads the implementation of a multidisciplinary ASP involving three infectious diseases clinicians (approximately 2 full-time equivalents), a microbiologist and a clinical pharmacist. Its key activities include the dissemination of local infection treatment protocols, generation of antimicrobial consumption and resistance reports, postprescription validation of restricted antibiotics, automatic 7-day stop orders, ad hoc clinical consultations, and weekly multidisciplinary meetings as part of a structured support model for clinical departments. Engagement with departments with structured meetings (Hemato-oncology, Renal Transplant Unit, Orthopedic Surgery, Neurosurgery, Hepatobiliary Surgery, Plastic Surgery, Burns Unit, Vascular Surgery, Urology, Cardiac Surgery, and Thoracic Surgery) happen either weekly at the meetings, sporadically whenever advice on a specific case is required or as needed during the elaboration of protocols or quality improvement interventions. For departments without structured meetings, engagement with UL-PPCIRA is more focused on validation of restricted antibiotics and occasional advice on specific cases. Despite these efforts, the extent to which specific stewardship initiatives influence prescribing behavior remains largely unmeasured. Understanding how prescribers perceive and interact with ASPs is necessary for identifying areas for improvement. Moreover, such an assessment may provide a useful model for other institutions aiming to align ASP activities with clinical realities.

This study aims to assess physicians’ perceptions of the ASP through a structured questionnaire. Additionally, beyond its local relevance, this study aims to illustrate a feasible model for assessing prescribers’ perceptions of stewardship programs.

## Materials and methods

This was a cross-sectional study conducted between June and December 2024 at Hospital de São João, a tertiary university hospital in Northern Portugal with 1,200 beds. The institution is highly differentiated, with active bone marrow, kidney, and heart transplant programs, a dedicated hematology-oncology service, and an advanced intensive care unit that includes extracorporeal membrane oxygenation support.

The study population consisted of all the physicians involved in antibiotic prescribing decisions at the institution. A convenience sampling method was employed: all eligible physicians were invited to participate via institutional e-mail. Participation was encouraged through monthly reminder emails sent to all eligible physicians over the course of the study period.

Data were collected using an anonymized online questionnaire developed by the research team and adapted from a French AMS study.^6^ The questionnaire, administered through Microsoft Forms, was designed to capture participants’ experiences with, opinions on, and perceived usefulness of the various interventions of the hospital’s ASP, as well as to identify potential barriers and facilitators to adherence. It comprised 39 items grouped into six domains: (1) knowledge of antibiotics; (2) awareness of local antibiotic prescribing protocols; (3) use of antimicrobial consumption reports; (4) awareness of the restricted antibiotics program; (5) engagement with unscheduled consultations on antimicrobial therapy; and (6) participation in scheduled weekly multidisciplinary case reviews. The tool was specifically developed for this study and has not been formally validated. Before dissemination, it was piloted with four resident physicians from different specialties at the same institution (not included in the final sample) to assess clarity, relevance, and comprehensiveness. These participants were selected for convenience, based on availability. Feedback from the pilot informed refinements to wording and structure. Additionally, members of the hospital’s ASP reviewed the final version for content appropriateness. The complete questionnaire can be found in the supplementary materials.

Descriptive statistics were used to summarize participant responses. To explore potential differences across professional profiles, we performed subgroup analysis comparing specialists versus residents and medical versus surgical specialties. The outcomes selected for comparison included awareness of institutional protocols, appreciation of scheduled case discussions, awareness of antibiotic restriction measures, and interest in receiving feedback on antimicrobial consumption or resistance data. For this exploratory analysis, selected variables were dichotomized to allow construction of 2 × 2 contingency tables. Differences between subgroups were tested using Pearson’s *χ* ^2^ test or Fisher’s exact test when expected cell counts were below 5. A two-sided *P* value < .05 was considered statistically significant. Missing data were handled using pairwise deletion, excluding only the specific data points that were missing for each analysis. Data analysis for descriptive statistics was conducted using Microsoft Excel. Fisher’s exact and *χ* ^2^ tests were calculated with MedCalc Software Ltd (Version 23.4.1).^7^

This study received prior approval from the hospital’s ethics committee. Informed consent was obtained from all participants after providing detailed information about the study objectives, procedures, potential risks, and benefits. Data protection measures included anonymization of all responses and secure storage of data, accessible only to members of the research team. Participation was voluntary, and respondents could withdraw at any time. The study was also conducted in compliance with the General Data Protection Regulation (GDPR).

## Results

Out of 1,539 surveys sent through institutional e-mail, 156 responses were received. Two were excluded because the respondents indicated they were not involved in antibiotic prescribing and were completing their general residency year. After exclusions, 154 valid responses were included, yielding an approximate 10% response rate. Most respondents were female (64.3%) and held specialist positions (75.3%), primarily in medical specialties (64.9%) (Table 1). All predefined age groups were represented, with the highest proportion of respondents aged 25 to 35 years (34.4%).

**Table 1.** Respondents’ characteristics (*N* = 154)

Characteristics	*N* (%)
**Age group**	
25–35	53 (34.4)
36–45	31 (20.1)
46–55	35 (22.7)
56 or more	35 (22.7)
**Sex**	
Female	99 (64.3)
Male	55 (35.7)
**Professional differentiation**	
Specialists	116 (75.3)
Residents	37 (24.0)
Missing data	1 (0.7)
**Specialties**	
Medical	100 (64.9)
Surgical	52 (33.8)
Missing data	2 (0.3)

### Antibiotic knowledge

Most respondents reported being able to manage simple infections only (59.6%). In terms of self-assessed knowledge, responses were predominantly classified as good (48.1%) or sufficient (37.0%) (Table 2A).

**Table 2.** Summary of survey responses by domain (*N* = 154)

A. Self-assessed knowledge and confidence in antibiotic use		
Question	Category	*N* (%)
Confidence in antibiotic prescribing	Confident and comfortable in choosing antibiotics.	56 (35.9)
	Confident in simple cases only.	93 (59.6)
	Not confident.	5 (3.2)
Self-rated knowledge	Very good	14 (9.1)
	Good	74 (48.1)
	Sufficient	57 (37.0)
	Insufficient	9 (5.8)

B. **Awareness and frequency of use of institutional protocols**		
Question	Response	*N* (%)
Are there institutional protocols for the management of infectious diseases and antimicrobial prescribing?	Yes	121 (78.6)
	No	1 (0.6)
	I don’t know	32 (20.8)
How often do you consult these protocols?	Never	36 (23.5)
	Rarely	67 (43.5)
	Monthly	40 (26.0)
	Weekly	7 (4.5)
	More than once a week	4 (2.6)

C. **Awareness of restrictive measures**		
Question	Response	*N* (%)
Are restrictive prescribing measures implemented in your department?	Yes	69 (44.8)
	No	36 (23.4)
	I don’t know	49 (31.8)
If yes: Are you aware of which antibiotics are included in the restricted list?*	Yes	26 (37.7)
	No	13 (18.8)
	I’m unsure	30 (43.5)
* Only respondents who answered yes to the first question were directed to this question.		

D. **Clinical Situations in which respondents seek unscheduled support from the AMS team**		
Clinical situation		N* (%)
Management of infections with which the clinician is unfamiliar.		75 (47.8)
Request for expert consultation.		73 (46.5)
Alternative antibiotic choice when first-line agents are not viable		45 (28.7)
Adaptation of therapy based on microbiological results.		33 (21.0)
Choice of antibiotic duration.		30 (19.1)
No support usually requested.		34 (21.7)
Empirical antibiotic selection.		18 (11.5)
Treatment of special populations		18 (11.5)
Clarification of antibiotic dosing.		11 (7.0)
Transition from intravenous to oral therapy.		2 (1.3)
*Respondents were allowed multiple answers for this question.		

E. Participation in scheduled discussions on antimicrobial prescribing		
Question	Response	N (%)
Are there scheduled meetings in your department for guidance on antimicrobial prescribing?	Yes	39 (25.3)
	No	98 (63.6)
	I don’t know	17 (11.0)
If yes: Do you consider the duration of these meetings appropriate?*	Yes	37 (94.9)
	No, too long and detailed	0 (0.0)
	No, too short and general	2 (5.1)
Do you consider the frequency of these meetings appropriate?*	Yes	38 (97.4)
	No	1 (2.6)
	Not applicable	0 (0.0)
* Only respondents who answered yes to the first question in this section were directed to this question		

F. Perceived usefulness of interventions and suggestions for improvement		
Question	Response	N* (%)
Which interventions do you consider most useful?	Availability of protocols.	112 (72.7)
	Unscheduled support to specialties (phone/email.)	87 (56.5)
	Regular meetings for clinical case discussions.	75 (48.7)
	Feedback on antimicrobial consumption and local epidemiological data.	71 (46.1)
	Automatic stop program (e.g., 7-day empirical or 24-hour surgical prophylaxis).	34 (22.1)
	Restricted antibiotics program (authorization requirement).	22 (14.3)
Which resources would you like to see reinforced or implemented in your department?	Direct inclusion of antibiotic recommendations in susceptibility reports	68 (44.2)
	Direct contact when antibiotic therapy could be optimized	61 (39.6)
	Direct contact when the isolated agent is resistant to the prescribed antibiotic	41 (26.6)
	Direct contact when the isolated agent is multidrug-resistant	41 (26.6)
	Educational sessions on antibiotic use in common infections	41 (26.)
	Dissemination of protocols	34 (22.1)
	Intervention programs to improve prescribing	27 (17.5)
	Department-specific ASP liaison (local contact point)	26 (16.9)
	Training sessions on antimicrobial resistance	21 (13.6)
	Direct contact when a restricted antibiotic is inappropriately prescribed	18 (11.7)
	Narrow-spectrum-first antibiograms	16 (10.4)
	Regular clinical case discussions	17 (11.0)
	Regular meetings to discuss ASP projects and improvement initiatives	8 (5.2)
* Respondents were allowed multiple answers for these questions		

### Protocols

Most respondents were aware of the existence of institutional protocols for antimicrobial prescribing (Table 2B). However, the frequency of use of protocols was generally low. Among those who rarely or never used the protocols, the most reported reasons included difficulty in locating the documents in the hospital’s shared folders (35.7%), reliance on preexisting knowledge (18.8%), lack of time (13.6%), and a preference for national or international guidelines (9.7%).

### Restrictive measures

Most respondents (55.2%) were not aware of the existence of postprescription validation of restricted antibiotics (Table 2C), having answered “no” or “I don’t know” when asked if restrictive measures were in place. Additionally, awareness of which antibiotics were restricted was low. As for the impact of these restrictive measures on clinical practice, most respondents (69.5%) agreed or strongly agreed that the measures influenced their prescribing practices, while 17.3% were neutral or disagreed.

### Unscheduled consultations

Situations prompting unscheduled consultation with the AMS team varied, with the most common reasons being managing unfamiliar infections and seeking expert opinion (Table 2D).

Regarding the frequency of unscheduled contact with the ASP team, 14.9% of respondents reported never requesting support, 51.3% rarely, 22.1% monthly, 9.7% weekly, and 1.9% more than once per week.

### Scheduled weekly multidisciplinary meetings

Only 25.3% of respondents reported having scheduled case-based discussions on antimicrobial prescription (Table 2E). Of those with scheduled meetings, most considered their duration and frequency adequate. Regarding the usefulness of these consultations, the vast majority (37 out of 39) found them beneficial for their clinical practice.

### Overview of interventions

When asked about the most useful AMS interventions, respondents most frequently selected the availability of protocols, unscheduled support for specialties, and regular case discussions. Regarding preferred resources for reinforcement or implementation, the most chosen were the inclusion of prescription recommendations in susceptibility reports, direct contact when antibiotic therapy could be improved, and structured training sessions on antimicrobial prescription (Table 2F).

### Feedback of data on antimicrobial use or antimicrobial resistance

A total of 129 respondents (83.8%) expressed interest in receiving regular updates on antimicrobial consumption within their unit, while 10 (6.5%) were not interested, and 15 (9.7%) reported no opinion. Interest in receiving periodic information on local AMR patterns was reported by 142 respondents (92.2%), whereas 3 (2.0%) were not interested, and 9 (5.8%) had no defined opinion.

### Subgroup analysis

No statistically significant differences were observed between specialists and residents regarding awareness of restrictive antibiotic measures (*χ* ^2^(1) = .58, *P* = .45) or frequency of protocol use (*χ* ^2^(1) = 0.39, *P* = .53). Similarly, no significant differences were found between medical and surgical specialties in awareness of restrictive measures (*χ* ^2^(1) = 0.02, *P* = .89) or frequency of protocol use (*χ* ^2^(1) = 1.02, *P* = .31).

There were also no significant differences between specialists and residents in the perceived usefulness of scheduled multidisciplinary case discussions (*χ* ^2^(1) = .05, *P* = .81), interest in receiving feedback on antimicrobial consumption (*χ* ^2^(1) = 0.86, *P* = .35), or interest in receiving local resistance data (*χ* ^2^(1) = .45, *P* = .50). Likewise, no significant differences were observed between medical and surgical specialties in interest in receiving feedback on antimicrobial consumption (*χ* ^2^(1) = .79, *P* = .37) or resistance patterns (*χ* ^2^(1) = 0.51, *P* = .47).

However, medical and surgical specialties differed significantly in how useful they considered scheduled case discussions, with surgical specialties selecting this intervention more frequently (77.4%) than medical specialties (40.8%), *χ* ^2^(1) = 16.06, *P* < .001.

## Discussion

This study explored how physicians perceive and engage with AMS activities at a tertiary hospital. Despite a modest response rate, the sample included a wide range of specialties and seniority levels, offering insight into AMS integration across the institution.

Four key findings emerged: (1) institutional protocols are underused, mainly due to accessibility issues; (2) regular weekly multidisciplinary case discussions are well received and could be expanded; (3) awareness of the hospital’s restrictive antibiotic policy is limited; and (4) there is high demand for resistance and consumption data among prescribers. Below, we discuss each of these findings and possible actions.

Most respondents knew protocols existed but used them infrequently, often preferring national guidelines or relying on self-knowledge. This is consistent with findings from the French national survey our study was based on,^6^ where 76.7% of prescribers relied on guidelines issued by academic societies compared to 61.5% who used local hospital protocols. Additionally, a study among general practitioners in Germany^8^ reported that only 39% frequently used antibiotic therapy guidelines.

The most cited barrier in our study was poor accessibility. Environmental restructuring,^9^ such as creating a centralized and clearly labeled section that facilitates consultation, either by placing it more visibly on the intranet homepage or by integrating it into the electronic health record system, could help improve usage. Although the latter option would require dialog with the IT department and potentially the software provider, even simple interface improvements may assist in overcoming this barrier. Lack of time, a frequent response by prescribers, may also be tackled by improving accessibility.

Some respondents also pointed toward barriers related to reflective motivation^9^: preference for national/international documents or self-knowledge, concerns on whether the protocols had been updated according to the most recent evidence and a perception of misalignment between the protocols and the clinical questions. It is therefore likely that improving accessibility should be complemented with a multidimensional approach including education (clarifying the elaboration methodology and update process, providing local epidemiological data and highlighting differences between local and international guidelines), persuasion (providing data feedback on adhesion to protocols and including nudges to incentivize protocol use in the electronic health records), enablement (structuring protocols to align more closely aligned with clinicians’ decision-making and covering special populations, providing summaries or practical algorithms and including front-line clinicians in protocol design) and modeling (including recognized experts in the elaboration and endorsement of protocols).^9^

Scheduled case discussions were viewed positively by respondents who had them within their departments, with the vast majority indicating that the frequency, duration, and clinical usefulness of these meetings were adequate. These results may support expanding scheduled discussions to more departments. While many AMS tools are automated or digital, the value placed on face-to-face, case-based discussions shows that clinicians still rely on personalized, real-time input for complex decisions regarding individual patients. Unscheduled consultations were less frequent and usually occurred when physicians faced difficult cases, which may reflect appropriate, case-driven use of the service rather than systematic integration into routine clinical practice.

Subgroup analyses showed no statistically significant differences between residents and specialists or between medical and surgical teams regarding awareness of protocols, awareness of restrictive measures, or interest in receiving feedback data. The only statistically significant finding was that surgical specialties more frequently valued scheduled multidisciplinary discussions, likely reflecting their greater routine exposure to these meetings at our institution.

Awareness of the hospital’s restrictive antibiotic policies was limited. Even among those who knew such measures were in place, many were unsure which antibiotics were restricted. While most respondents agreed that these policies influence their prescribing decisions, the results suggest that communication around their scope and rationale could be improved. At the institutional level, restrictive antibiotic prescribing policies follow national AMS guidelines.^4^ All prescriptions for quinolones, carbapenems, ceftolozane/tazobactam, ceftazidime/avibactam, colistin, vancomycin, linezolid, daptomycin, and newly authorized antibiotics require mandatory justification in the electronic prescription system and are reviewed within 72 hours by a designated ASP physician. In cases of insufficient or unclear justification, direct discussion with the ASP team may occur. One possible approach to improve awareness of this restriction process could include sharing an updated and simplified list of restricted antibiotics with clinical departments, accompanied by brief explanations. Opportunities to reinforce this information might include departmental meetings or routine AMS interactions.

Interest in receiving regular feedback on aggregated AMR and consumption data was high. These findings support the potential value of systematic feedback strategies. However, previous research suggests that even when such institutional data are available, they may not substantially influence prescribing behaviors.^10,11^ For example, in a recent study,^11^ only 26.9% of hospitalists reported consulting antibiograms more than once a month, and many indicated that their empiric choices were largely unaffected by the reported susceptibility thresholds. This suggests that aggregated resistance data must be presented with interpretative guidance relevant to common clinical scenarios. Integration into existing prescribing tools may also facilitate their use.

The most valued ASP interventions included access to protocols, expert support, and therapeutic guidance embedded in microbiology reports. In general, prescribers tend to support educational and supportive interventions over restrictive measures.^6^ Additionally, team dynamics and autonomy influence prescribing decisions, supporting the idea that ASP strategies should be adapted to local contexts.^5^

This study has several limitations. Firstly, the response rate was low, which may introduce selection bias, particularly if those more engaged with stewardship were more likely to respond. However, physician surveys are known to often yield low participation rates. A meta-analysis of 11 physician surveys^12^ found a mean response rate of 18.9%, with some as low as 7.4%, depending on topic and engagement strategy. In addition, the denominator may have overestimated the number of eligible prescribers, as the distribution list included all institutional physician e-mail addresses, some of which may have been inactive due to short-term contracts, residency rotations, retirement, or staff turnover. Secondly, the questionnaire was not formally validated, although it was adapted from a previously published tool^6^ and piloted locally. In addition, responses were self-reported and may be subject to recall bias. Finally, as a single-center, cross-sectional study, the findings may not be generalizable and do not capture changes over time.

Despite these limitations, the study offers relevant insights for assessing ASP quality as perceived by the medical population and improving implementation. The inclusion of respondents from various specialties and training levels provides a broad perspective on prescribing behavior.

In conclusion, this survey identified four priority areas for strengthening AMS at our institution: improving the accessibility of institutional protocols by reorganizing intranet access paths or exploring quick-access integration into clinical software; maintaining and potentially expanding scheduled multidisciplinary case discussions by offering regular meetings to additional departments; enhancing communication around restricted antibiotics through simplified summaries of restrictive policies and reinforced messaging during routine AMS activities; and providing regular feedback on antimicrobial consumption and resistance through tools that pair data with clear interpretative guidance. Beyond its local relevance, this study illustrates how prescriber perspectives can guide the development of more context-sensitive stewardship strategies. Understanding how clinicians interact with AMS components can inform targeted, effective interventions. Future research could evaluate these interventions longitudinally and examine behavioral drivers influencing prescribing across clinical areas.

## Supporting information

10.1017/ash.2025.10263.sm001 Valois et al. supplementary material Valois et al. supplementary material

## Data Availability

The datasets presented in this article are not readily available due to GDPR restrictions.

## Supplementary material

The supplementary material for this article can be found at https://doi.org/10.1017/ash.2025.10263.
