# Antimicrobial stewardship at a tertiary center in Portugal: insights from prescribers – ERRATUM

**DOI:** 10.1017/ash.2026.10386

**Published:** 2026-04-22

**Authors:** André Valois, Mariana Salvado de Morais, Lúcia Ribeiro Dias, Francisco Almeida, Mariana Guedes, Paulo Andrade, Nuno Rocha-Pereira

**Affiliations:** 1 Department of Biomedicine, Faculty of Medicine, University of Porto, Porto, Portugal; 2 Clinical Pharmacology Unit, São João Local Health Unit (ULS São João), Porto, Portugal; 3 Internal Medicine Department, São José Local Health Unit (ULS de São José), Lisbon, Portugal; 4 Department of Medicine, Faculty of Medicine, University of Porto, Porto, Portugal; 5 Infectious Diseases Department, São João Local Health Unit (ULS São João), Porto, Portugal; 6 Infection and Antimicrobial Resistance Control and Prevention Unit, Hospital Epidemiology Centre, São João Local Health Unit (ULS São João), Porto, Portugal; 7 Department of Public Health and Forensic Sciences, and Medical Education, University of Porto, Porto, Portugal

This article was originally published with incorrect numbering of affiliations. Affiliations 5 and 6 were interchanged during production, which resulted in incorrect institutional attribution for several authors.

The errors have been corrected and the online HTML and PDF versions updated.

The publisher apologises for this error.
