# Supplementary material for: Antimicrobial stewardship at a tertiary center in Portugal: insights from prescribers
Source: Antimicrob Steward Healthc Epidemiol. 2025 Dec 22;6(1):e8. doi: 10.1017/ash.2025.10263 (PMC12766505; doi:10.1017/ash.2025.10263)
Supplement: Valois et al. supplementary material [file S2732494X25102635sup001.docx]

Survey Questionnaire on Antimicrobial Stewardship Practices and Perceptions

## Age range

- 25–35
- 36–45
- 46–55
- ≥ 56

## Sex

- Female
- Male

## Level of training

- Hospital Assistant / Senior Hospital Assistant
- Medical Intern in Specialty Training
- Medical Intern in General Training

## Specialty (if applicable)

______________________

## Unit where you work

______________________

## Do you prescribe or participate in antimicrobial therapy decisions for inpatients?

- Yes
- No

## How do you assess yourself regarding infectious disease treatment and antibiotic prescription?

- Comfortable and confident in choosing antibiotics
- Able to manage simple cases, but have difficulty with complex ones
- Not confident, with doubts regarding antibiotic prescription

## When in doubt about antibiotic prescription, what sources do you usually consult? (Select up to 3)

- Colleagues in your unit
- Internal Medicine Department
- Infectious Diseases Department
- Microbiology Department
- Infection Prevention and Control Unit (UPCIRA)
- Guidelines / Bibliography
- Hospital protocols
- Other

## How would you rate your knowledge of antibiotic therapy?

- Very good
- Good
- Adequate
- Insufficient

## To what extent do you agree with the following statements when choosing antimicrobial therapy?

I consider the patient’s characteristics and comorbidities

- Strongly disagree
- Disagree
- Neutral
- Agree
- Strongly agree

I consider disease severity

- Strongly disagree
- Disagree
- Neutral
- Agree
- Strongly agree

I consider the infectious focus

- Strongly disagree
- Disagree
- Neutral
- Agree
- Strongly agree

I consider environmental/epidemiological impact

- Strongly disagree
- Disagree
- Neutral
- Agree
- Strongly agree

I choose the narrowest possible spectrum

- Strongly disagree
- Disagree
- Neutral
- Agree
- Strongly agree

I prefer a defensive approach

- Strongly disagree
- Disagree
- Neutral
- Agree
- Strongly agree

I prescribe the shortest effective duration

- Strongly disagree
- Disagree
- Neutral
- Agree
- Strongly agree

I switch to oral antibiotics as soon as possible

- Strongly disagree
- Disagree
- Neutral
- Agree
- Strongly agree

I follow national and local guidelines/protocols

- Strongly disagree
- Disagree
- Neutral
- Agree
- Strongly agree

I narrow antibiotic spectrum after isolation

- Strongly disagree
- Disagree
- Neutral
- Agree
- Strongly agree

## Are there established protocols for infectious disease management and antimicrobial prescription at CHSJ?

- Yes
- No
- Don't know

## Where are these protocols available?

- Online (Intranet)
- Institutional email
- Posters in the department
- Physical copies in the library
- Don’t know
- Other

## How often do you consult these protocols?

- Never
- Rarely
- Monthly
- Weekly
- More than once per week

## If rarely used, why?

- Difficult to find
- Protocols don’t address my clinical doubts
- I disagree with the guidelines
- I know them well and don’t need to consult often
- I prefer national/international guidelines
- Protocols are outdated
- Lack of time
- Other

## Main reasons for consulting the protocols:

- Empirical therapy choice
- Management of uncommon infections
- Alternatives in case of allergy/resistance
- Adjusting therapy based on microbiology results
- Defining treatment duration
- Choosing route of administration
- Other

## When do you find it hard to follow protocols?

- When they don’t match clinical severity
- When they don’t consider patient specifics
- When patient already received antibiotics elsewhere
- When they don’t align with local practice
- When they don’t match specialty-specific practices
- When in doubt about efficacy of first-line treatment
- I do not find it difficult
- Other

## Would you like to receive regular reports on antibiotic use in your unit?

- Yes
- No
- Don't know

## Would you like to receive regular reports on resistance patterns in your unit?

- Yes
- No
- Don't know

## Are there measures in your unit restricting antimicrobial prescriptions?

- Yes
- No
- Don't know

## If yes, which ones? (Select up to 3)

- Mandatory indication justification
- Mandatory treatment duration definition
- Automatic discontinuation (e.g., after 7 days or 24h for surgical prophylaxis)
- Pre-authorization by UPCIRA or Pharmacy
- Other

## Do you know which antibiotics are included in the restricted list?

- Yes
- No
- Unsure

## When a restricted antibiotic is denied, is the reason communicated to you?

- Yes
- No
- Not applicable

## When your prescription is denied, can you discuss it with a UPCIRA physician?

- Yes
- No
- Not applicable

## Do current restriction measures influence your clinical practice?

- Strongly disagree
- Disagree
- Neutral
- Agree
- Strongly agree

## In which situations do you contact UPCIRA (outside scheduled meetings)? (Select up to 3)

- Empirical therapy choice
- Alternative in case of resistance/allergy
- Adjusting therapy based on microbiology
- Unfamiliar infections
- Dose clarification
- Duration of therapy
- IV to oral switch
- Expert opinion
- Special populations (pregnancy, renal/hepatic failure, immunosuppressed)
- I do not seek support

## How do you contact UPCIRA? (Select up to 3)

- Phone
- Email
- In person
- Not applicable

## How often do you contact UPCIRA without a scheduled meeting?

- Never
- Rarely
- Monthly
- Weekly
- More than once per week

## Is contacting UPCIRA accessible?

- Strongly disagree
- Disagree
- Neutral
- Agree
- Strongly agree

## Are there scheduled antimicrobial stewardship meetings in your department?

- Yes
- No
- Don't know

## Is the duration of these meetings appropriate?

- Yes
- No (too long or too short)

## Is the frequency appropriate?

- Yes
- No
- Not applicable

## If not, what would be the ideal frequency?

- Once a week
- More than once a week
- Less than once a week

## Are these meetings useful to your clinical practice?

- Yes
- No
- Not applicable

## If yes, what are the main benefits? (Select up to 3)

- Improve patient outcomes
- Multidisciplinary approach
- Reduce hospital-wide resistance
- Limit resistance in treated patients
- Optimize dosing, duration, route
- Reduce side effects (e.g. C. difficile, nephrotoxicity)
- Improve surgical infection decisions
- Reduce hospital stay
- Cost reduction
- Increased confidence and safety in prescriptions

## If no, why are these meetings not useful?

____________________________________________________

## Regarding UPCIRA's antimicrobial management interventions, to what extent do you agree with the following?

I receive the support I need

- Strongly disagree
- Disagree
- Neutral
- Agree
- Strongly agree

I would like more decision support tools

- Strongly disagree
- Disagree
- Neutral
- Agree
- Strongly agree

They limit prescriber autonomy

- Strongly disagree
- Disagree
- Neutral
- Agree
- Strongly agree

They improve patient outcomes

- Strongly disagree
- Disagree
- Neutral
- Agree
- Strongly agree

They hinder therapeutic decisions

- Strongly disagree
- Disagree
- Neutral
- Agree
- Strongly agree

They reduce length of stay

- Strongly disagree
- Disagree
- Neutral
- Agree
- Strongly agree

They reduce antibiotic-related complications

- Strongly disagree
- Disagree
- Neutral
- Agree
- Strongly agree

They help prevent resistance

- Strongly disagree
- Disagree
- Neutral
- Agree
- Strongly agree

They reduce hospitalization costs

- Strongly disagree
- Disagree
- Neutral
- Agree
- Strongly agree

They benefit attending physicians

- Strongly disagree
- Disagree
- Neutral
- Agree
- Strongly agree

They improve department functioning

- Strongly disagree
- Disagree
- Neutral
- Agree
- Strongly agree

They improve broader medical service functioning

- Strongly disagree
- Disagree
- Neutral
- Agree
- Strongly agree

They improve surgical service functioning

- Strongly disagree
- Disagree
- Neutral
- Agree
- Strongly agree

## Which interventions do you consider most useful? (Select up to 3)

- Antibiotic use and resistance feedback
- Protocol availability
- On-demand support to specialties (phone/email)
- Scheduled clinical case discussions (PAPA)
- Restricted antibiotic approval program
- Automatic stop orders

## Which resources should be enhanced or implemented? (Select up to 3)

- Direct prescription advice in susceptibility tests
- Contact when resistant/multidrug-resistant pathogens are isolated
- Contact when antibiotic therapy can be improved
- Contact when inappropriate restricted antibiotic is prescribed
- Antibiograms showing only narrow-spectrum options
- Educational sessions on common infections
- Educational sessions on resistance
- Intervention programs to improve prescribing
- Service-specific PAPA liaisons
- Protocol dissemination
- Case discussion meetings
- Feedback and planning meetings

## What suggestions do you have to improve UPCIRA’s activities?

____________________________________________________
